# Tumor cell metabolic reprogramming and hypoxic immunosuppression: driving carcinogenesis to metastatic colonization

**DOI:** 10.3389/fimmu.2023.1325360

**Published:** 2024-01-16

**Authors:** Theodora Katopodi, Savvas Petanidis, Doxakis Anestakis, Charalampos Charalampidis, Ioanna Chatziprodromidou, George Floros, Panagiotis Eskitzis, Paul Zarogoulidis, Charilaos Koulouris, Christina Sevva, Konstantinos Papadopoulos, Marios Dagher, Vasileios Alexandros Karakousis, Nikolaos Varsamis, Vasiliki Theodorou, Chrysi Maria Mystakidou, Konstantinos Vlassopoulos, Stylianos Kosmidis, Nikolaos Iason Katsios, Konstantinos Farmakis, Christoforos Kosmidis

**Affiliations:** ^1^ Department of Medicine, Laboratory of Medical Biology and Genetics, Aristotle University of Thessaloniki, Thessaloniki, Greece; ^2^ Department of Pulmonology, I.M. Sechenov First Moscow State Medical University, Moscow, Russia; ^3^ Department of Anatomy, Medical School, University of Cyprus, Nicosia, Cyprus; ^4^ Department of Public Health, Medical School, University of Patra, Patra, Greece; ^5^ Department of Electrical and Computer Engineering, University of Thessaly, Volos, Greece; ^6^ Department of Obstetrics, University of Western Macedonia, Kozani, Greece; ^7^ Third Department of Surgery, “AHEPA” University Hospital, Aristotle University of Thessaloniki, Thessaloniki, Greece; ^8^ Department of Surgery, Interbalkan Medical Center, Thessaloniki, Greece; ^9^ Department of Medicine, Faculty of Health Sciences, Aristotle University of Thessaloniki, Thessaloniki, Greece; ^10^ Department of Medicine, Medical University of Plovdiv, Plovdiv, Bulgaria; ^11^ Medical School, Faculty of Health Sciences, University of Ioannina, Ioannina, Greece; ^12^ Pediatric Surgery Clinic, General Hospital of Thessaloniki “G. Gennimatas”, Aristotle University of Thessaloniki, Thessaloniki, Greece

**Keywords:** tumor metabolism, reprogramming, immunosuppression, metastasis, hypoxia

## Abstract

A significant factor in the antitumor immune response is the increased metabolic reprogramming of immunological and malignant cells. Increasing data points to the fact that cancer metabolism affects not just cancer signaling, which is essential for maintaining carcinogenesis and survival, but also the expression of immune cells and immune-related factors such as lactate, PGE2, arginine, IDO, which regulate the antitumor immune signaling mechanism. In reality, this energetic interaction between the immune system and the tumor results in metabolic competition in the tumor ecosystem, limiting the amount of nutrients available and causing microenvironmental acidosis, which impairs the ability of immune cells to operate. More intriguingly, different types of immune cells use metabolic reprogramming to keep the body and self in a state of homeostasis. The process of immune cell proliferation, differentiation, and performance of effector functions, which is crucial to the immune response, are currently being linked to metabolic reprogramming. Here, we cover the regulation of the antitumor immune response by metabolic reprogramming in cancer cells and immune cells as well as potential strategies for metabolic pathway targeting in the context of anticancer immunotherapy. We also discuss prospective immunotherapy-metabolic intervention combinations that might be utilized to maximize the effectiveness of current immunotherapy regimes.

## Introduction

The metabolic reprogramming of cancer cells is necessary for the genesis and development of tumors (1, 2). In order to fulfill the increased energetic and biosynthetic demand as well as reduce oxidative stress, cancer cells autonomously change their flow through several metabolic pathways (3). These changes are necessary for cancer cells to proliferate and survive. Otto Warburg, (1931, Nobel Prize in Medicine) developed insights that served as the foundation for current understanding of cancer metabolism (1, 4). It is commonly known that in aerobic circumstances, normal cells obtain their energy first from glycolysis occurring in the cytosol, then from mitochondrial oxidative phosphorylation (OXPHOS) (5). When oxygen is in low supply, glycolysis provides the cells with energy rather than the oxygen-dependent mitochondrial metabolism. However, compared to normal cells, tumors have a distinct metabolic pattern. Aerobic glycolysis (Warburg effect) refers to the reality that cancer cells choose to complete glycolysis in the cytosol even when oxygen is present (6). In addition to the glycolytic characteristic, cancer cells also undergo significant changes in cellular lipid composition, fatty acid production, oxidation, and other aspects of lipid metabolism (7). The metabolism of amino acids is commonly changed in tumor cells during carcinogenesis and cancer development (8). A vastly acidic, nutrient-deficient, and hypoxic tumor microenvironment (TME) is the result of all of these cell-intrinsic metabolic abnormalities, which exacerbates the metabolic rewiring events in cancer cells and immunocytes of the tumor microenvironment TME (9). Insufficient glucose production prevents T cells from undergoing glycolysis, resulting in anergy in which T cells are unable to increase cytokine release and proliferation in response to stimulation (10). By activating AMPK (AMP-activated protein kinase) while suppressing mTOR (mammalian target of rapamycin) and HIF-1 (Hypoxia-inducible factor 1), glucose deprivation also raises the ratio of AMP to ATP, which promotes the differentiation of CD4+ T cells into immunosuppressive Tregs rather than CD4+ effector T cells (Teffs) (11). This encourages the production of more anti-inflammatory M2 macrophages than M1 macrophages. Additionally, the buildup of lactate in the TME prevents CD8+ T cells from proliferating and secreting cytokines by obstructing MAPK signaling, and also causes exhaustion in these cells as a result of lactic acidosis (12). By promoting ARG1 (arginase 1) expression, lactate is absorbed by macrophages to cause differentiation into the immunosuppressive M2 macrophages. Immunocytes’ immunological activity can be impacted by signaling programs through metabolism (13). In this context, co-stimulation and antigen recognition through the T cell receptor (TCR) are two essential signals that are strongly related to metabolic reprogramming in activated T cells (14). When both are present, glucose transporters and glycolysis-related enzymes are increased, and T cells are stimulated with proliferation and the production of cytokines like IL-2. In T cells, glycolysis also controls translation and transcription (15). According to research, when lactate dehydrogenase (LDH) in CD4+ T cells is decreased in order to limit glucose intake, less acetyl-CoA is produced, which leads to insufficient acetylation of histones at the location of IFN-γ genes, reducing IFN-γ production (16). Through a sequence of enzymes, the glycolysis intermediate fructose-6-phosphate (Fru6P) can be employed in the hypoxia-driven hexosamine biosynthetic pathway (HBP) to produce uridine diphosphate N-acetylglucosamine (UDP-GlcNAc) (17). For post-translational modifications such as O-GlcNAcylation of proteins crucial for immune cell differentiation and proliferation, UDP-GlcNAc is utilized (18). Lipid metabolism also has a significant impact on immune cells in addition to glycolysis. Lipids are necessary for the manufacture of cellular membranes, which is necessary for growth and proliferation (19). The enzyme responsible for controlling the rate of fatty acid production is acetyl-CoA carboxylase 1 (ACC1). Lack of ACC1 prevents T lymphocytes from expanding and persisting during the antigen-specific response (20). Fatty acid oxidation (FAO), in addition to the fatty acid production stated above, can also have an impact on immune cells. Memory CD8+T cells have higher levels of the FAO’s restricting enzyme, carnitine palmitoyltransferase 1A (CPT1A), which helps memory cells to survive after removing antigens and respond quickly to antigens when they are rechallenged with them (21). By preserving a fluid cell membrane, which drives TCR clustering, cholesterol metabolism benefits T cell activation. ACAT1 induces the creation of cholesterol ester from acyl-CoA and free cholesterols for the storing of free cholesterols (22). Increased intracellular cholesterol levels of tumor-infiltrating lymphocytes (TILs) in melanoma are caused by pharmacologically or genetically inhibiting ACAT1, which enhances immunological responses (23). The creation of proteins and nucleotides, which accelerates cellular development, is fuelled by amino acids. Numerous cell types can employ amino acids to inhibit the immune system due to their importance in metabolism (24). Through promoting the production of catabolic enzymes that create necessary amino acids, such as ARG1 and IDO (indoleamine 2,3-dioxygenase), TAMs (tumor-associated macrophages), MDSCs (myeloid-derived suppressor cells), and immunotolerant DCs (dendritic cells), trigger the suppression of TILs (25). For instance, MDSCs prevent T cells from synthesizing enough cysteine, which is essential for tumor fighting immune defenses (26). In conclusion, as metabolism controls the actions of both tumor cells and immune cells, therapeutic regimes for cancer patients may result from detailed targeting of metabolism.

## Metabolic reprogramming of tumor and immune cells

**Figure 1:**
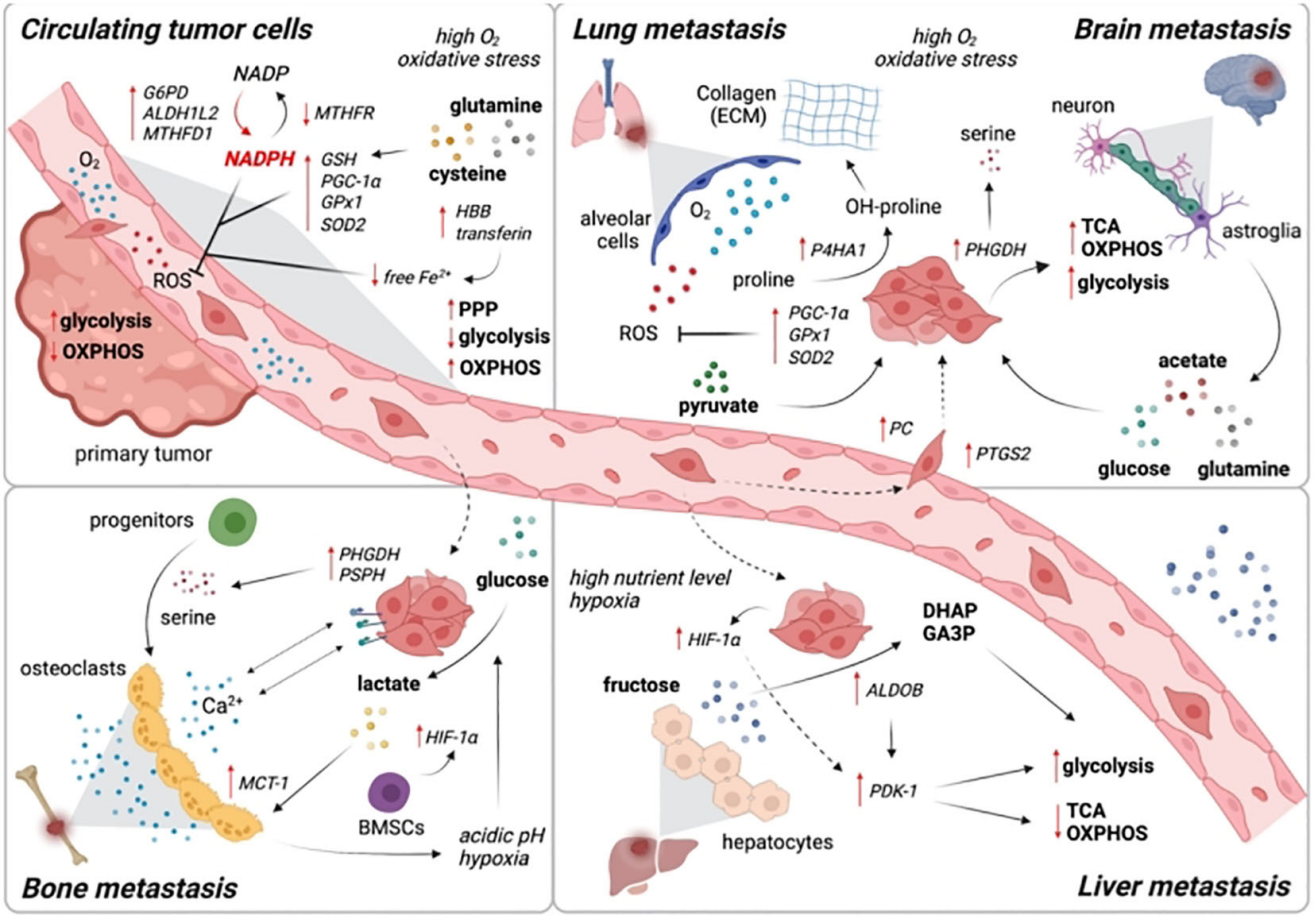
The metabolic reprogramming to OXPHOS controls the dormancy of cancer cells. When cancer cells from primary tumors reach distant organs, they frequently go into dormancy. The transition from anabolism to catabolism, which powers this latent state, gives cancer cells the redox power and energy they need to live in the hostile environment. This metabolic transition is driven by the activation of ATG3 (Autophagy-related gene 3), ATG7 (Autophagy-related gene 7), and p62 which upregulates autophagy. This dormant state, is linked to metabolic reprogramming toward lipid and protein catabolism and OXPHOS activation. Cancer cells must reprogram their metabolism toward anabolism in order to emerge from the latent state and achieve the high rates of proliferation required for metastatic colonization and the creation of overt metastasis. Reproduced with permission from Ref (27). Copyright 2021, Elsevier.

Reprogramming of the energy metabolism, which promotes rapid cell division and proliferation through modifications to the energy metabolism, has been identified as an emerging trait of cancer (**Figure 1**) (28). Tumor is a diverse and heterogeneous disease with a complicated metabolic pattern due to the variability of its cells and structure (29). For instance, in hypoxic settings, tumor cells often generate pyruvate via the glycolysis route, which results in the production of lactic acid rather than Acetyl-CoA, which is then converted to make ATP in the mitochondria (30).

The well-known aerobic glycolysis (Warburg effect) is nevertheless preferred by tumor cells to produce ATP, even when there is enough oxygen available (3). At the same time, tumor cells employ glutamine, serine, arginine, fatty acids, and lipid compounds to boost their own proliferation in addition to breaking down glucose to produce ATP (1). Surprisingly depending on the amount of external nutrients and the type of stress present, tumor cells will select alternative metabolic pathways to create ATP and biological macromolecules for their own needs (31). For instance, under the stress condition of nutrient deprivation, such as that of glucose or glutamine, tumor cells activate the oncogene c-Myc to support the survival of tumor cells by controlling the expression of metabolic enzymes like PHGDH (phosphoglycerate dehydrogenase), PSAT1 (phosphoserine aminotransferase 1), PSPH (phosphoserine phosphatase), and other metabolic enzymes in the serine synthesis pathway, promoting the *de novo* synthesis of serine, and sustaining redox homeostasis (32). Additionally, in order to supply energy for their own survival and bio-macromolecules such as fatty acids to support their own survival, tumor cells synthesize acetyl-CoA by consuming the nominal two-carbon fatty acid (acetoacetate) under hypoxic or nutrient-deficient stress circumstances (33). Similar to this, tumor cell metabolites created by the breakdown of ketone bodies may enter the TCA cycle to supply ATP for cell survival (34). As a result, tumor cells have a complicated and variable metabolic modes. Depending on their environment, they will select the best metabolic mode to ensure their survival (35).

## Tumor-induced immune metabolism and metabolic rewiring of immune cells

**Figure 2:**
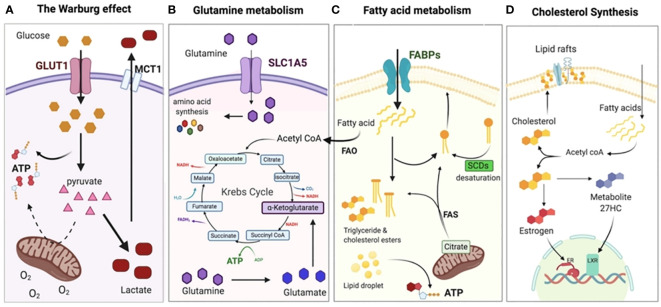
Metabolic adaptation of tumor cells. **(A)** Even when there is a sufficient supply of oxygen, cancer cells often adopt the Warburg effect or aerobic glycolysis, relying on glycolysis rather than OXPHOS for the production of ATP. As a result, the pyruvate is changed into lactate and discharged outside of the cell, where it acidifies the TME and creates an immunosuppressive environment. **(B)** The Krebs (TCA cycle and other anabolic processes, such as the creation of nucleotides and other amino acids), shift the dependency of cancer cells on glutamine. In addition, glutamine plays a crucial role in the production of glutathione, which is essential for chemo-resistance. **(C)** To supplement glycolysis for energy, fatty acid oxidation (FAO) and fatty acid synthesis (FAS) are both increased to supplement glycolysis for energy and to provide the necessary membrane components for accelerated cell development. **(D)** In order to signal and maintain the structure of their membranes, cancer cells need to synthesize cholesterol in order to expand and metastasize. Reproduced with permission from Ref (37). Copyright 2020, MDPI.

Numerous immune cells, including macrophages, neutrophils, monocytes, eosinophils, basophils, lymphocytes, and natural killer cells, build up the immune system mechanism. When the body is in an inactive resting state, these cells are dormant (14). However, when the body is triggered by an infection, an inflammatory reaction, or another external agent, these cells are immediately aroused and react (36). It’s interesting to note that tumor and immune cells also have these sophisticated and diversified metabolic processes (**Figure 2**). Recent research indicates that the energy requirements of immune cells in their resting and active states varies significantly (38). T cells are one type of immune cell that performs a variety of tasks, including removing infections and eliminating tumors. According to various activation levels, T cells will exhibit entirely diverse metabolic pathways. For instance, naive T cells’ metabolism is essentially static and exhibits little proliferative activity, necessitating the maintenance of just the barest minimums of food intake, glycolysis rate, and biosynthesis (39). OXPHOS produces the majority of ATP. It manifests as a metabolic activation state, boosting food absorption, rising glycolysis rate, and synthesizing buildup of protein, lipid, and nucleotide once an external stimulus has activated an effector T cell (40). The mitochondrial oxygen consumption decreases at the same time, allowing T cells to finally multiply and give rise to offspring cells that can carry out effective killing functions (41). It’s interesting to note that the metabolic pattern of memory T cells is comparable to that of naive T cells, with maintenance of basic food intake, a slower rate of glycolysis, and reliance on OXPHOS to produce ATP (42).

Additionally, the primary source of energy for active neutrophils, M1 macrophages, and iNOS-expressed DCs is glycolysis. Although oxidative phosphorylation is mostly used by DCs for energy metabolism when they are at rest, glycolysis plays a significant role in the activation of DCs (43). In the meantime, the lipid metabolism is altered and its activity is impacted by DC activation. Additionally, the primary metabolic routes of neutrophils include pentose phosphate pathways and aerobic glycolysis (44). Numerous crucial neutrophil activities, including respiratory burst and chemo-taxis, are controlled by glycolysis (45). Furthermore, glycolysis and mitochondrial metabolism are improved following the stimulation of B lymphocytes by LPS or an antigen. However, the primary metabolic process in activated B lymphocytes is glycolysis. Tregs and M2 macrophage, on the other hand, primarily rely on OXPHOS from FAO to supply energy (46). The development of various immune cell subgroups is influenced by the metabolic rewiring inside the TME. For instance, in the TME, glucose restriction prevents T cells from secreting more cytokines and from proliferating in response to stimulation, resulting in anergy (47). Through the activation of AMPK and the inhibition of mTOR and HIF-1α, glucose deprivation also increases the ratio of AMP : ATP. This promotes the generation of anti-inflammatory M2 macrophage phenotypes over M1 macrophages and supports the differentiation of CD4^+^ T cells into immunosuppressive Tregs rather than CD4^+^ Teffs (38). Furthermore, lactate buildup in the TME causes lactic acidosis, which makes CD8^+^ T cells anergic and prevents them from proliferating or secreting cytokines by blocking MAPK signaling (48). Macrophages absorb lactate and differentiate into immunosuppressive cells whereas DCs and myeloid cells differentiate into to tumor-associated DCs (TADCs) and MDSCs respectively (40). Hence, investigating immune cell metabolic reprogramming and the impact of rewiring the metabolic activities of immune cells would aid in comprehending the fundamentals of immune response and its regulation mechanism.

## Metabolic hypoxic immunosuppression

**Figure 3:**
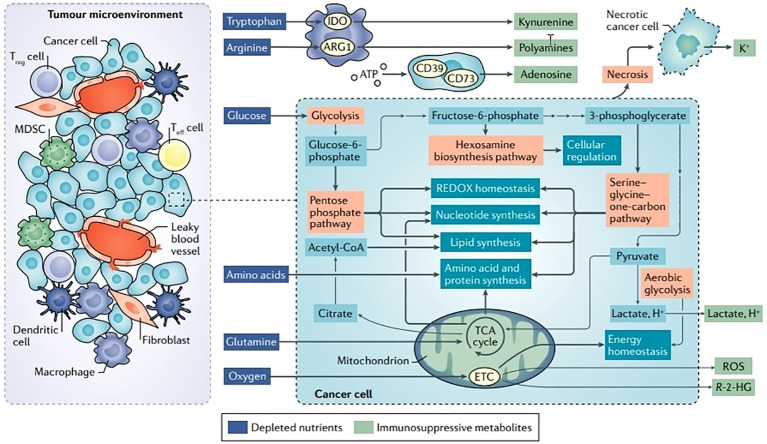
Disturbances in TME metabolism and cancer cell metabolism. Energy is produced for dormant, differentiated cells in the mitochondria by the oxidation of substances such as glucose, amino acids, and fatty acids using the TCA cycle and the electron transport chain (ETC). However, cells upregulate an alternate mechanism for glucose consumption known as aerobic glycolysis during times of enhanced proliferation, such as following immunological activation or malignant transformation. Aerobic glycolysis, although being less effective in producing ATP, enables faster glucose metabolism, effective carbon dioxide removal, and NAD+ regeneration while preserving mitochondrial enzymatic activity for anabolic activities. Glutamine is the principal source of nitrogen required for amino acid and nucleic acid synthesis. Ectoenzymes, such as IDO, ARG1, and CD73, which deplete nutrients, are expressed by a large number of cells in the TME. Reproduced with permission from Ref (49). Copyright 2020, Springer Nature.

Recent research data indicate that T cells in the TME, particularly Tregs, display a distinct metabolic immunosuppressive phenotype (**Figure 3**) (24). Due to an increased rate of fatty acid production, Tregs that infiltrate tumors in mouse tumor models accumulate intracellular lipids (50). This reveals that both glycolytic and oxidative metabolism result in the growth of Tregs, suggesting that comparative advantage in glucose absorption may cause fatty acid production in intratumoral Tregs (51). Inhibition of CD4+ Tregs’ ability to suppress the immune system by TLR8 (Toll like receptor 8) is also correlated with glucose metabolism in ovarian cancer (52). Genes and proteins linked to glucose metabolism are down-regulated in CD4+ Tregs when TLR8 is activated, along with a reduction in glucose absorption and glycolysis (53). In addition, Weinberg et al. demonstrated the need of mitochondrial respiratory chain complex III for the immunosuppressive action of Tregs. In mouse models, Treg-specific ablation of mitochondrial complex III accelerates the development of a deadly inflammatory disease without affecting the cellular quantity of Tregs (54). Furthermore, mice lacking mitochondrial complex III specifically in Tregs show a loss in the ability to suppress T cells due to enhanced DNA methylation and increased levels of relevant metabolites like succinate and 2-hydroxyglutarate (2-HG), which block the TET (ten-eleven translocation) family of DNA demethylases (55). Cancer cells can also release succinate into their microenvironment and activate succinate receptor (SUCNR1) signaling to polarize macrophages into TAMs and promote metastasis (56). Additionally, FABP5 (fatty acid binding protein 5) regulates mitochondrial integrity, which has an impact on Treg activity. OXPHOS and lipid metabolism are compromised when FABP5 is inhibited in Tregs, and the production of cardiolipin, which is crucial for maintaining mitochondrial integrity, is decreased (57). Damaged mitochondria trigger the release of mitochondrial DNA into the cytoplasm, which leads to type I IFN signaling that is reliant on cGAS-STING (cyclic GMP–AMP synthase–stimulator of interferon genes), which increases the production of the regulatory cytokine IL-10 and encourages Treg immunosuppressive activity (58). Tregs are impacted by acid metabolism in addition to glycolysis, fatty acid metabolism, and mitochondria. Serine feeds into the 1CMet (one-carbon metabolic network), which is essential for Teff responses, and encourages glutathione (GSH) production (59).

Kurniawan et al. precisely eliminated the catalytic subunit of glutamate cysteine ligase (Gclc) in mouse Tregs, resulting in Treg-specific GSH depletion, in order to study the impact of serine metabolism in Tregs (60). They discovered that GSH-deficient Tregs exhibit increased serine metabolism and reduced expression of FoxP3. Additionally, mice with Gclc deficiency that are unique to Treg have improved anti-tumor responses. Gclc-deficient Tregs restore FOXP3 expression and immunosuppressive capacity when serine supply is blocked by giving the mice foods poor in serine. Tumor-inhibiting CD8^+^ T cells participate in anti-tumor responses. Metabolic modification modifies CD8^+^ T cell activity in the TME. Tumor cells demonstrate a preferential intake of glucose from the perspective of glycolysis, which limits the availability of glucose to anti-tumor T cells (61). For instance, the methyltransferase eEZH2 (enhancer Of zeste 2 polycomb repressive complex 2 subunit) is restricted in expression by the overexpression of the microRNAs miR-101 and miR-26a in ovarian cancer, which lowers the amount of glucose available to T cells (62). The development of anti-tumor immune responses depends on aerobic glycolysis. Hexokinase 2 (HK2), a vital glycolytic enzyme, can be stabilized by the action of NF-B inducing kinase (NIK). Inhibiting glycolysis and HK2 levels, NIK deletion prevents CD8^+^ T cells in the TME from performing their effector roles (63). Gemta et al. discovered that enolase 1, a crucial glycolytic enzyme, downregulates its activity in CD8^+^TILs and causes abnormalities in CD8^+^TILs’ glycolytic metabolism in both human and mouse melanomas. Fatty acid metabolism also has a significant impact on T cells in addition to glycolysis (64). The PPAR (peroxisome proliferator-activated receptor signaling) and fatty acid catabolism are strengthened in the hypoglycemia and anoxic TME regions of mouse melanoma models by CD8^+^ TILs, preserving the effector activity of TILs. The immunotherapeutic impact of melanoma can be enhanced by stimulating CD8^+^ TILs’ fatty acid metabolism. From an amino acid standpoint, glutamine is becoming into a targetable metabolite in tumor treatment (65). For glutamine from the microenvironment, Teffs and tumor cells compete in the mouse triple-negative breast cancer (TNBC) model. Glutaminase, a vital enzyme for glutamine metabolism, is specifically deleted from tumor cells, which boosts T cell activation and improves anti-tumor immune responses (66). Additionally, the glutamine transporter inhibitor V-9302, which does not influence anti-tumor T cells but can specifically block glutamine uptake in TNBC cells, offers a viable treatment approach for TNBC (67). The therapeutic impact of glutamine antagonism on T cells and tumor cells is therefore distinct. In tumor-bearing mice, glutamine antagonism slows cancer cells’ glycolytic and oxidative metabolism, up-regulates oxidative metabolism, and causes antitumor T lymphocytes to develop a long-lasting and highly activated phenotype (68). Tumor immunotherapy uses the difference in metabolic response to glutamine antagonism between T lymphocytes and tumor cells as a metabolic checkpoint. T cells also depend on the shape and metabolism of the mitochondria (69). Through MYC-regulated pathways, the oxygen-deprived TME increases mitochondrial fragmentation and lowers ATP synthesis to cause T cell fatigue (exhaustion) (70).

## Metabolic competition between immune and tumor cells in the TME

**Figure 4:**
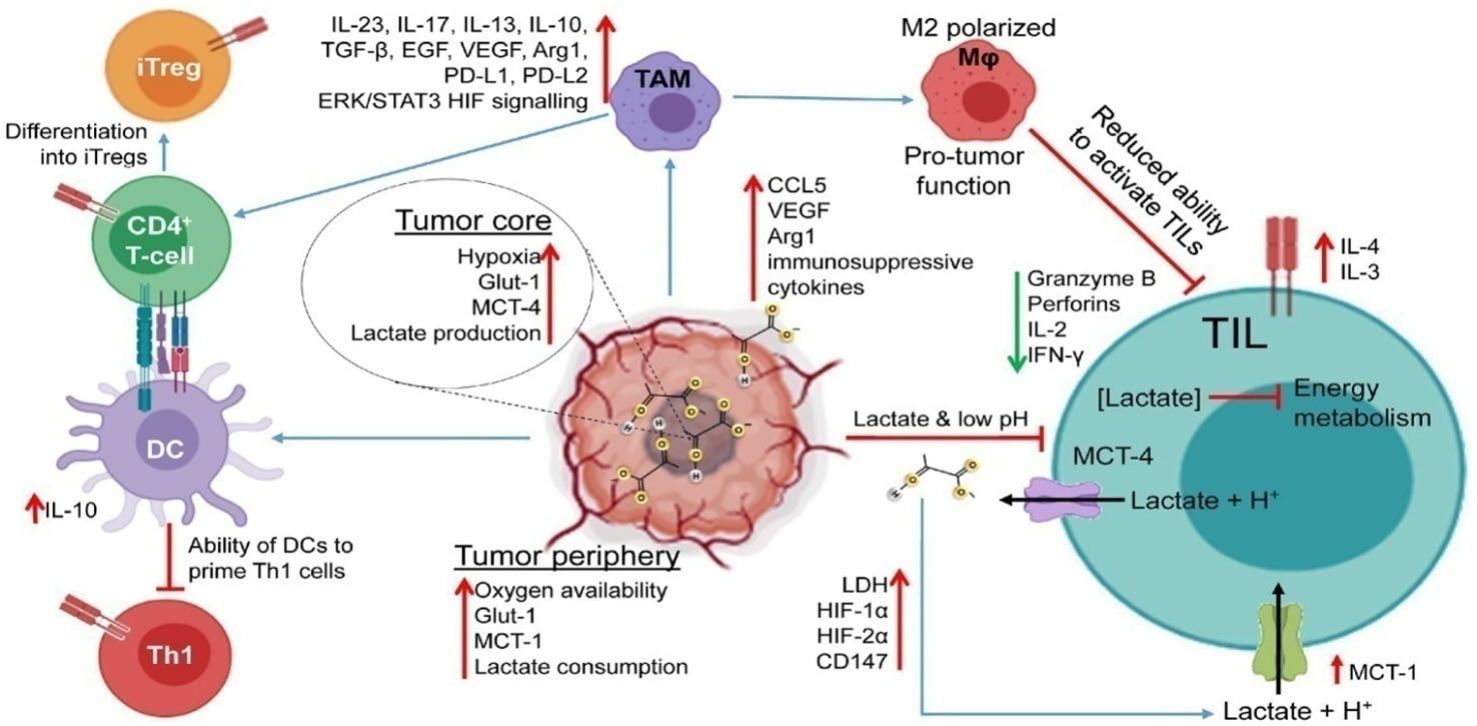
Impaired immune response in the TME. Lactic acid accumulation in the TME interferes with immunological processes, impairing the T-cell anti-tumor response. The TME becomes acidic due to hypoxia in the tumor core, excessive glucose intake, and increased expression of LDH and lactate transporters. By inhibiting energy metabolism, upregulating inhibitory receptors (like PD-1), interfering with TCR signaling, and producing immunosuppressive cytokines, enzymes, and signaling proteins (like IL-4, IL-10, CCL5, TGF, and VEGF), increased TME acidity impairs the function of TILs. Additionally, it prevents DCs from activating Th1 cells and tilts TAM polarization toward the M2 phenotype. These M2-like TAMs aid tumor cells in evading the TIL onslaught because they are incapable of phagocytizing tumor cells. Reproduced with permission from Ref (72). Copyright 2022, Elsevier.

Cancer cells are not the only cells that undergo metabolic transitions; activated T cells, Treg cells, macrophages, and other rapidly reproducing cells also exhibit these changes (57). The nutrient on which tumor cells need the most, glucose, is also a critical energy source required for the activation, development, and functionality of immune cells (10). Different levels and kinds of immune cell infiltration are present along with the TME. TILs need nutrients from the TME to enable differentiation and proliferation, much like cancer cells do (71). Even when there are adequate tumor antigens for T cells to detect, data show that tumors suppress the activity of tumor-infiltrating T cells by competitive absorption of glucose (**Figure 4**) (73).

In fact, a number of recent investigations have shown that the glycolytic activities of cancer cells may limit the amount of glucose that TIL T cells can consume, leading to T-cell depletion and immunological escape (74). Actually, a significant increase in the tumor’s microenvironmental consumption of glucose will unavoidably impair T cell function by altering T cell metabolic processes. Glycolytic metabolites have been shown in the literature to negatively impact immune system function (75). The competitive uptake of amino acids, glutamine, fatty acids, and other metabolites or growth factors by tumor cells and immune cells, as well as the expression of corresponding transporters on the cell surface, are still significant factors affecting the functioning of immune cells, although the damage to T cell function is caused by the competitive uptake of glucose under acidic TME conditions (76). On top of that, the TME has high amounts of lactate and low pH, hypoxia, and high levels of ROS, all of which contribute to the growth of cancer and immune escape. Cooperation and competition between cell populations of the TME supports tumor proliferation, progression, metastasis, and immune evasion (77). Cellular heterogeneity leads to metabolic heterogeneity because metabolic programs within the tumor are dependent not only on the TME cellular composition but also on cell states, location, and nutrient availability. In addition to driving metabolic plasticity of cancer cells, altered nutrients and signals in the TME can lead to metabolic immune suppression of effector cells and promote regulatory immune cells (78). This causes chemoresistance, and reduction of tumor therapy efficacy. In order to avoid the damaging effects of metabolic competition between the tumor and the immune system, addressing these metabolic pathways in malignancies may be a potential strategy to increase the immunogenicity of tumors (79). However, further research is required to test this theory in both current and upcoming preclinical models.

## Metabolic fitness

**Figure 5:**
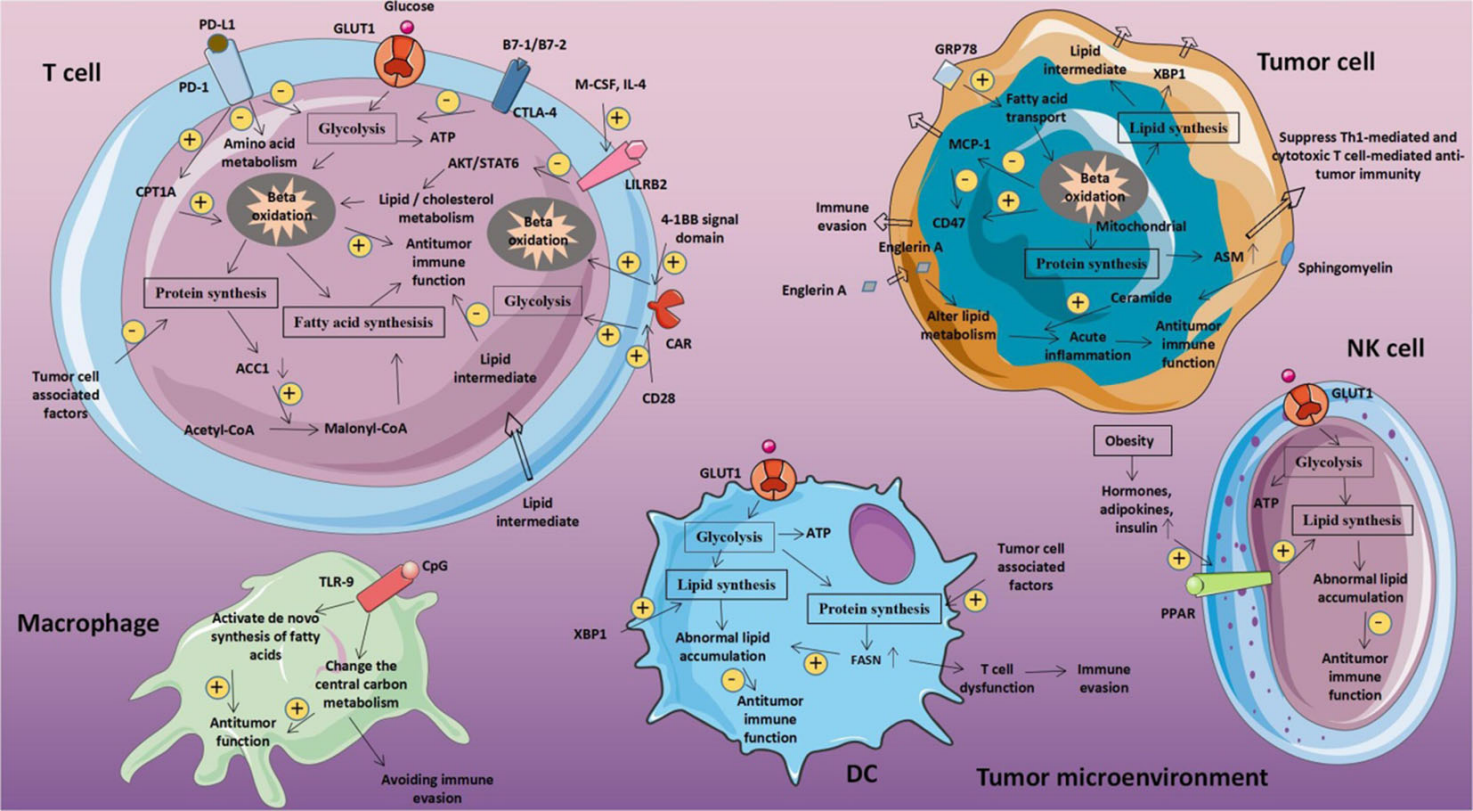
Metabolic fitness triggers abnormal glucose metabolism in tumor and immune cells respectively. The tumor microenvironment may be impacted by metabolic disorders linked to glucose in tumor cells. T cell glucose levels decline as a result of tumor cells competitively absorbing glucose from the extracellular environment. This reduces T cell energy source, prevents production, and impairs T cell activity. Reproduced with permission from Ref (81). Copyright 2020, Frontiers.

The TME’s lack of nutrients and oxygen puts it at a metabolic disadvantage and causes immune cells that infiltrate tumors to become exhausted (80). As a result, immune cells like CD8+ T cells must develop and maintain metabolic fitness in order to respond to unfavorable metabolic circumstances (**Figure 5**). Acylglycerol kinase (AGK) positively regulates PTEN (phosphatase and tensin homolog) phosphorylation and inhibits PTEN’s phosphatase activity in CD8+ T cells when PTEN accumulates on the plasma membrane through the mediation of TCR and CD28, promoting activation of PI3K-mTOR modulating glycolysis, and controlling anti-tumor activity (82). In terms of functionality, AGK is essential for preserving CD8+ T cells’ metabolic fitness in the TME (83). The proliferation of CD8+ T lymphocytes and their capacity to fight tumors are both compromised by the deletion of the lipid kinase acylglycerol kinase (AGK). AGK promotes the glycolytic and functional fitness of CD8+ T cells by inactivating PTEN and boosting mTOR activity, thereby promoting antitumor activity (84). The prognosis of individuals with EL4 (mouse lymphoblastic lymphoma) T lymphomas that overexpress ovalbumin is improved by S-2-HG therapy. The mitochondrial biogenesis and morphology are linked to the functional activity of CD8+ T cells in addition to kinases and metabolites. PGC1, which is essential for mitochondrial assembly and is reduced in CD8+ T cells in human melanoma, leading to intratumoral T cell dysfunction (85). PGC1 expression and mitochondrial biosynthesis support CD8^+^ T lymphocytes’ nutritional requirements and maintain metabolic fitness in order to create prolonged anti-tumor response (86).

In the fight against cancer, CD8+ T lymphocytes with effector and memory functions are both crucial. Given the long-lasting anti-tumor immunity offered by memory CD8+ T cells, the conversion of effector CD8+ T cells into memory CD8+ T cells is crucial to halting tumor development (87). The fact that serine/threonine kinase AKT inhibition prevents T cells from surviving throughout this metamorphosis suggests that metabolic activity is crucial in controlling how CD8+ T cells store memories (88). PCK1 (phosphoenolpyruvate carboxykinase 1) is also linked to memory CD8+ T cells in addition to AKT: Memory CD8+ T cells were discovered to up-regulate PCK1, which increased glycogen. gluconeogenesis is the process of creation (89). The conversion of glycogen into glucose-6-phosphate is then catabolized. to start the PPP (pentose phosphate pathway), which will result in NADPH is necessary to enhance GSH and oxidized ratio of glutathione and reduce ROS levels in storage CD8+T cell. Targeting the PCK1-glycogen-PPP axis thus raises ROS levels and prevents the development of memory CD8+ T cells (90). In addition to intracellular control, environmental factors like nutrition can significantly impact metabolic health and antitumor T cell activity. A low-protein diet, as opposed to a diet high in carbohydrates, slows the growth of tumors in mice, according to research (91). A low-protein diet stimulates RIG1 (retinoic acid inducible gene 1) and IRE1 (inositol-requiring enzyme 1) signaling to cause the unfolded protein response and cytokine generation in cancer cells, increasing CD8+ T cell antineoplastic effector activities (92). It has been documented that CD8+ T cells with long-term metabolic fitness are essential for the most effective response to anti-cancer therapy. The adoptive transfer of CD8+ T cells that have been grown ex vivo to develop metabolic fitness can help enhance tumor prognosis in addition to altering CD8+ T cells metabolism in vivo (93). In murine T cells, the high concentration of L-arginine in the culture media lowers glycolysis and increases OXPHOS, which leads to the development of the CD8+ central memory T cell phenotype. When such CD8+ T cells are adoptively transplanted into mice, survival advantage and good anti-tumor effector activity can be attained (94). On top of that, cholesterol biosynthesis and adoptive transfer methods of autologous T cells work together to control CD8+ T cells’ anti-tumor activity and metabolic fitness. A higher amount of plasma membrane cholesterol results in improved TCR signaling and encourages the development of synapses (95). ACAT-1 (acetyl-coA acetyltransferase-1) deletion enhances synaptic function in CD8+ T lymphocytes by up-regulating cholesterol biosynthesis enzymes and raising plasma membrane cholesterol levels (38). Furthermore, cholesterol induces tumor-infiltrating CD8+ T cell exhaustion in the TME by upregulated T cell expression of PD-1, 2B4, TIM-3, and LAG-3 (96). The adoptive transfer of mouse CD8+ T cells with a faulty form of ACAT-1 results in a better prognosis for melanoma tumors than wild-type CD8+ T cells. As a result, the ACAT inhibitor avasimibe demonstrated strong anti-tumor activity in a mouse melanoma model (97). Another element that affects T cell activity is hypoxia. In order to completely eradicate cancer cells, adoptively transplanted cytotoxic T lymphocytes are routinely cultivated with 20% oxygen. According to recent research data, CTLs that are grown under 1% oxygen exhibit increased granzyme-B production and improved cytolysis in response to B16 melanoma cells (98).

## Metabolic control of metastasis

**Figure 6:**
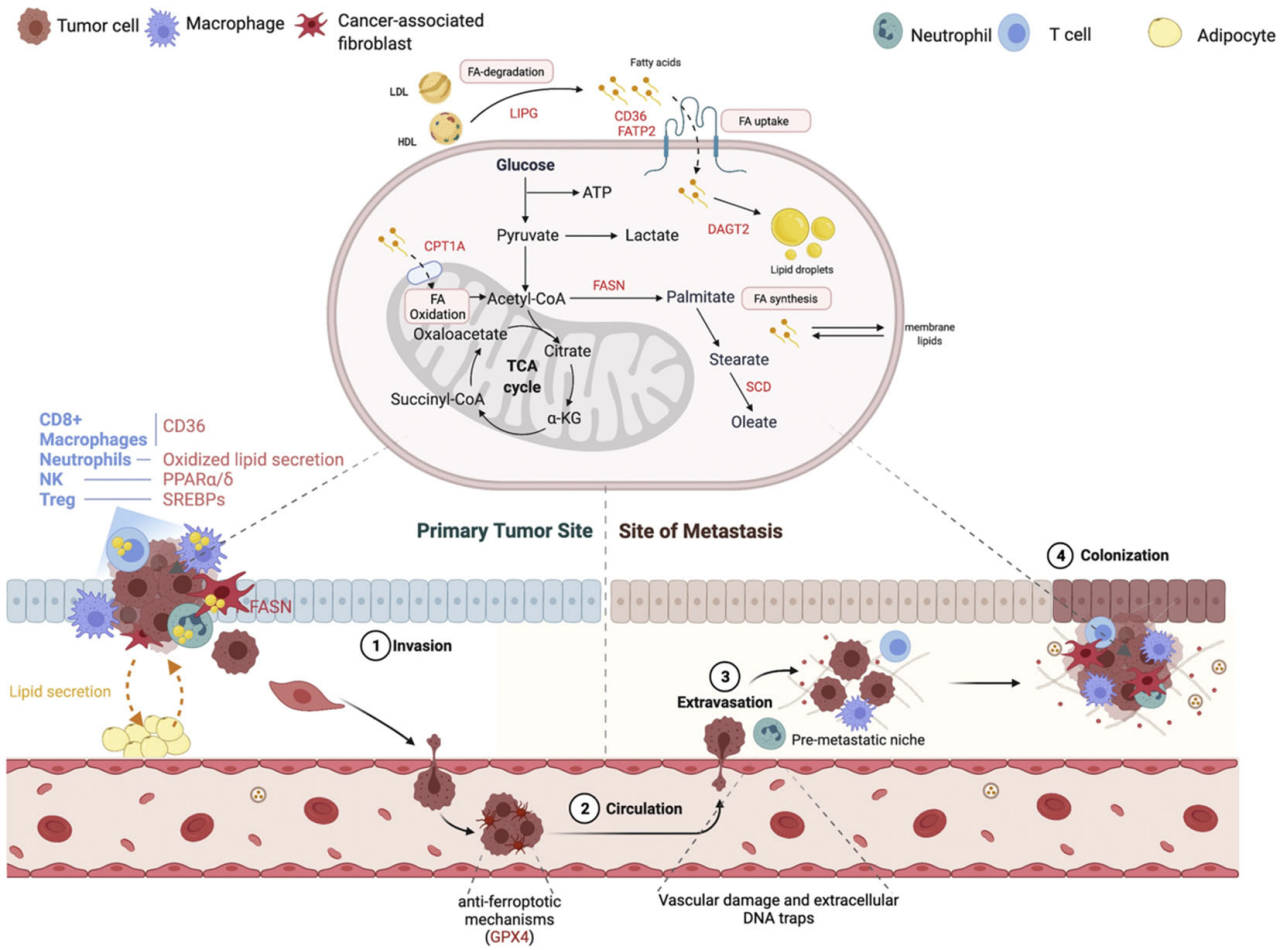
Changes in lipid metabolism that contribute to the metastatic cascade. At each stage of the metastatic cascade, the primary lipid-mediated mechanisms and genes are changed in the tumor cell and the tumor stroma. Reproduced with permission from Ref (102). Copyright 2021, Elsevier.

The study of metastasis, a key factor in the survival of cancer patients, has been increasingly popular in recent years as a subfield of cancer metabolism research (99). Deciphering the metabolic vulnerabilities of cells that metastasize and colonize distant places will be crucial for the scientific community because there are not many studies in this field (100). The process of metastatic spread of primary cancer cells to secondary locations is ineffective, and metabolic restrictions are now understood to be a hindrance to the ability of cancer cells to metastatic spread (101). The sequential multistep processes of metastasis, including invasion of the basement membrane and cell migration into the lymphatic or surrounding vasculature (intravasation), survival in the circulation and extravasation from the vasculature and colonization of secondary tumor sites, are all correlated with metabolic changes (**Figure 6**) (103). Intravasation is connected to a shift in the phenotypic of cancer cells from one that is proliferative to one that is invasive and migratory, which is frequently tied to EMT (epithelial-mesenchymal transition) and is, in part, controlled by TGF-dependent transcriptional alterations (104). The finding that cells with elevated MCT1 (monocarboxylate transporter 1) expression undergo metastasis inside a primary tumor is indicative of metabolic heterogeneity being connected to metastatic potential within the main tumor. The assessment of metabolic heterogeneity, which is causally related to metastatic potential, might be done using non-invasive technologies with spatial resolution (105). The cataloging of metabolic pathways that are not necessary for basic development but become crucial for metastasis will receive a lot of attention in the upcoming years. Numerous investigations have shown that mitochondrial activity is related to metastatic potential, perhaps as a result of the generation of ROS as signaling molecules (9fv). Loss of TIGAR (TP53 induced glycolysis regulatory phosphatase) may also lead to an increase in mitochondrial ROS and the possibility for metastatic spread. TGF (transforming growth factor), a major inducer of the EMT phenotype, can also boost ROS in the mitochondria (106). In addition to ROS, certain metabolites have been related to boosting EMT through their role as signaling molecules. For instance, research on fumarate hydratase (FH)-deficient tumors have shown that too much EMT phenotype may be amplified by fumarate through epigenetic modifications (107).

To reach and colonize remote areas, metastatic cells must endure in the lymphatic or vascular system. Cancer cells do not exist in an anabolic condition during their journey; rather, they transition into a catabolic state in order to survive the altering environment (108). Just now are efforts being made to overcome the particular metabolic constraints imposed by the circulatory and lymphatic systems. Oxidative stress is raised by loss of adhesion to the extracellular matrix. Cancer cells can die by stress-induced cell death, which is modifiable by clumping of cells, which causes hypoxia, resulting in a reduction of HIF1-mediated antioxidant stress (109). This may clarify why correlations between elevated HIF1 stabilization has a significant propensity for metastasis. Based on the unique nutritional availability in the new TME compared to the originating tumour site, the colonization at distant locations necessitates metabolic adaptation (110). Examining brain tumor metastases provides a telling illustration. Serine and fatty acids are two limiting nutrients in the TME of the brain; hence, breast cancer cells that infiltrate the brain have high expression of PHGDH to enable glucose-dependent serine and glycine synthesis (111). As a result, primary tumor development was unaffected by genetic and pharmaceutical reduction of PHGDH, while brain metastasis was reduced. In comparison to original tumors, metastatic breast cancer cells invading the brain rely more on *de novo* lipogenesis. Additionally, to overcome obstacles to anabolism in the new metastatic niche, metabolites with specific roles in collagen hydroxylation of the extracellular matrix and mTORC1 (mammalian target of rapamycin complex 1) signaling, such as pyruvate and serine, are needed (112).

## Metabolic targeting and immunotherapy

The use of metabolic treatment combined with immunotherapy to target tumor metabolism and control immune metabolism have shown promising results. In NSCLC (non-small cell lung cancer) the addition of pemetrexed to immunotherapy improved overall clinical survival (113). In particular, NSCLC, melanoma, renal cell carcinoma, and other malignancies may benefit more from combinational immunotherapy and specifically targeted metabolic inhibition. Targeting of AMPK, PI3K, IDO, lactate and adenosine pathways prevents deleterious metabolites from suppressing the immune response against cancer (114). These strategies, which synergize with immunotherapy, must be adapted to the type and stage of cancer and to the inter individual variability of drug response. This metabolic targeting can potentially change the TME and boost immune infiltration in cancers that have not responded well to immunotherapy, such as pancreatic, prostate, breast, and other cancers (39). For example pan-PI3K inhibitor (BKM120) has shown promising results when combined with immunotherapy in metastatic bladder cancer and melanoma. Results reveal increased lymphocyte infiltration and cytotoxic functions of CTLs through the synergistic action of anti-PD1 antibody with BMK120 to reduce tumor growth (115). In addition lactate metabolism inhibition by targeting MCTs (medium-chain triglycerides) or LDHA (lactate dehydrogenase A) improves the efficacy of immunotherapy. These include inhibitors such as 3-BrPA (3-brompyruvate), DCA (dichloroacetate) and AZD3965 currently tested in clinical trials for solid tumors, diffuse large B cell lymphoma and gliomas (116). Likewise, A2AR (adenosine 2A receptors) blockage on immune cells can be used as immunotherapy for treatment of refractory renal cell cancer (RCC) (117). In a similar manner, metformin is an antidiabetic medication that potentiates anti-Warburg effects on a variety of cancer types, including breast, colon, and lymphoma malignancies. Metformin pharmacologically activates AMPK. In fact, metformin modifies the energetic activity of cancer cells by blocking complex 1 of the electron transport chain, restricting the activity of the protumor isoform HK2, and downregulating the expression of HIF-1α and mTOR (118). In a phase I clinical study for metastatic melanoma (NCT03311308) and a phase II clinical trial for head and neck squamous carcinoma (NCT04414540), pembrolizumab and metformin are so coupled. Simultaneously, nivolumab and metformin combination therapy is being investigated for non-small-cell lung cancer (NCT03048500) (119). Inhibitors of additional metabolic enzymes in gliomas, such as glutaminase (GLS1) via CB-839, fatty acid synthase (FASN) via TVB-2640, isocitrate dehydrogenases (IDH) 1-2 via ivosidenib (AG-120) and vorasidenib (AG-881), and IDO-1 via indoximod (1-MT) and epacadostat (INCB024360) (120). Likewise, modulators of membrane-bound proteins, such as 5′-nucleotidase CD73 via α, β-methylene ADP (APCP), and the toll-like receptors (TLRs) 3–7/8 via the agonists poly (I:C) stabilized by lysine (poly-ICLC) and MEDI9197; (iii) inhibitors of arginase 1 in TAMs via molecules like CB-1158 (121). Finally, recent data indicates that CAR T cell signaling of certain co-receptor domains can modify T cell metabolism and stimulate CD8+ central memory T cells with higher fatty acid oxidation and respiratory capacity (122). These metabolic treatment strategies when combined with immunotherapy can make these tumors more susceptible to treatment.

## Future metabolic perspectives

**Figure 7:**
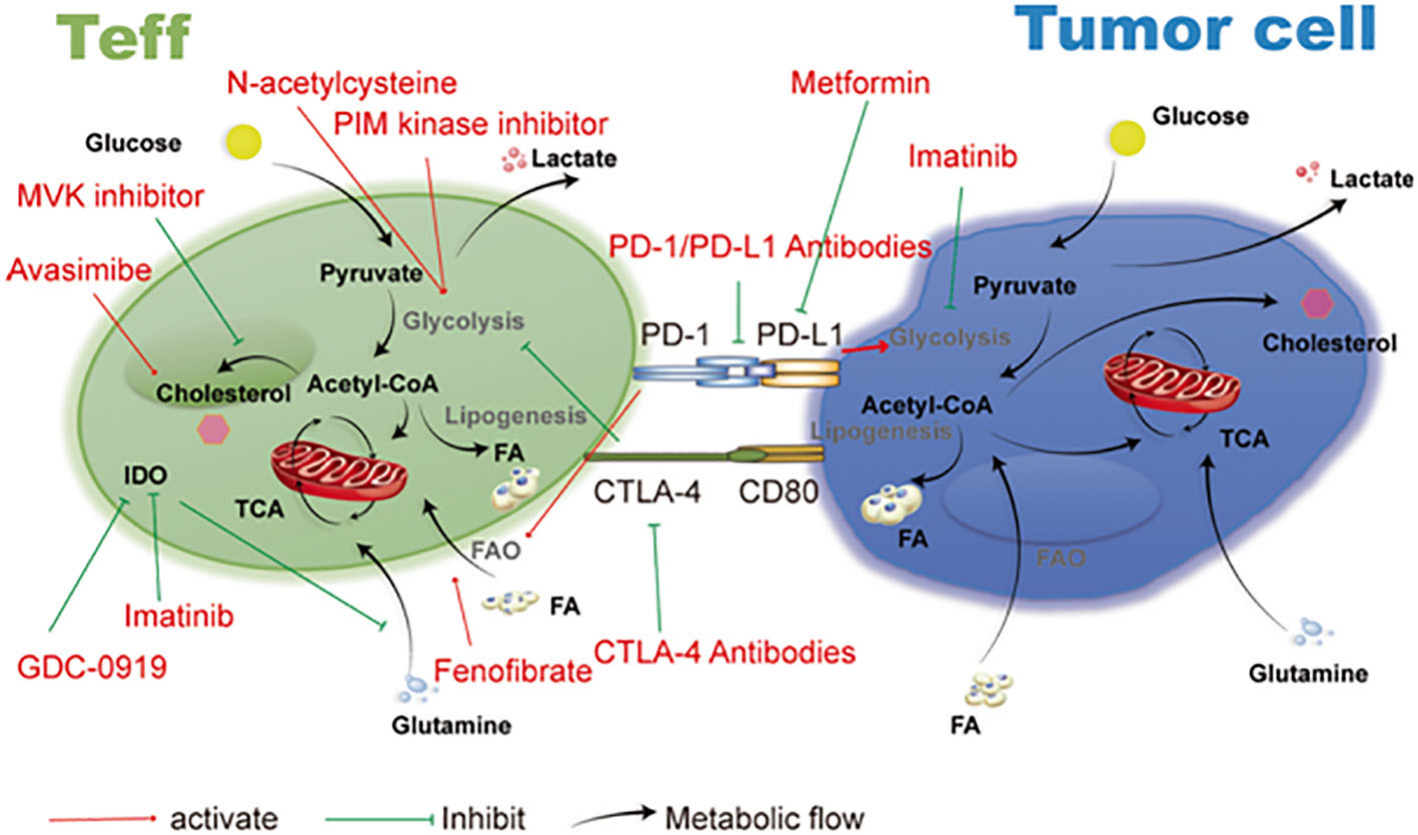
Therapeutic targets and cancer metabolism-blocking drugs. Immune cell activities are further inhibited by tumor cells’ competition with immune cells in the microenvironment for the nutrients needed for their own metabolism. Imatinib, metformin, and drugs that target the metabolic functions of T cells and tumor cells all have an important anti-tumor impact. Reproduced with permission from Ref (123). Copyright 2019, Springer Nature.

A deeper comprehension of the mechanisms causing metabolic interventions in immune and cancer cells may potentially point to novel therapeutic targets. For instance, it has been demonstrated that pharmaceutical intervention can influence the metabolic fitness and durability of the T cell (**Figure 7**) (124). Similar results were seen when T cells were treated with a PI3K inhibitor *in vitro*, leading to less differentiated cells with better *in vivo* persistence and antitumor activity in mice (125). This is related to the roles of AKT-mTOR signaling in promoting a terminally differentiated effector phenotype and increasing glycolytic flux upon T cell activation. The use of a 4-1BB co-stimulatory domain improves *in vivo* persistence by inducing mitochondrial biogenesis, OXPHOS, and subsequent memory T cell development, as opposed to the use of a CD28 domain, which enhances T cell glycolysis and effector differentiation (126). These findings suggested a connection between T cells’ intervening metabolic patterns and their role in the antitumor response. The discovery that rapamycin-induced mTORC1 inhibition increases the production of memory T cells following viral clearance provided an early indication of the critical function of metabolic control in activated T cells during a first immune response (127). During *in vitro* development of CD8^+^ T cells, the suppression of mTORC2-AKT signaling or glycolysis, which is the metabolic hallmark of CD8^+^ T effector cells might also give the cells a memory phenotype and boost their antitumor activity (128). Rapamycin, however, can reduce T lymphocyte activation and differentiation by inhibiting mTOR signaling, indicating that it has a potent immunosuppressive effect (129).Additionally, the rapamycin can suppress dendritic cells, promote the generation of regulatory T cells, and block CD8^+^ T cells, which all contribute to the immune system’s ability to fight cancer (130). Interestingly, AKT inhibitors can also metabolically modify both tumor-reactive and naïve TILs during *in vitro* growth, leading to a memory-like phenotype and enhanced anticancer efficacy after allogeneic transplantation into immune-deficient, myeloma-bearing animals (131). Similarly, the utilization of a PPARα (peroxisome proliferator-activated receptor alpha) agonist to improve the metabolic profile allows TILs to sustain effective anticancer activity in the TME even in the absence of oxygen and glucose (132). Furthermore, certain metabolic pathways in T cells can be targeted to improve their anti-tumor response. For example, inhibiting glycolysis or lactate production triggers inhibition of effector T cells. In addition, restricting glycolysis can limit terminal differentiation promote cell longevity and a memory phenotype (19). Likewise, inhibiting fatty acid synthesis could impair Th17 differentiation and promote Treg development. Furthermore, enhancing fatty acid oxidation could promote Treg or memory cell development. In a similar manner, blocking glutamine metabolism can inhibit T cell proliferation and effector T cell development (133). Finally, enhancing mitochondrial function through increasing mass, OXPHOS or altering mitochondrial dynamics can trigger T cell longevity. In conclusion, more research is required to better understand the mTOR pathway’s methods of action in various tumor cells and the accompanying tumor microenvironment, as well as to assess the overall therapeutic efficacy of mTOR inhibitors in the future.

It’s interesting to note that TILs, both naive and tumor-reactive, can undergo metabolic manipulation with AKT inhibitors during *in vitro* expansion, which results in a memory-like phenotype and increased antitumor activity when allogeneic transplanted into multiple myeloma-bearing mice with impaired immunity (134). However, with a markedly repressive impact on cell proliferation, inhibiting mTOR or the glycolytic pathway also promotes T cell differentiation towards naive and memory phenotypes (135). Additionally, despite being depleted of oxygen and glucose, TILs are still able to retain effective anticancer activity in the TME thanks to metabolic signature amplification employing a PPAR agonist (136). According to this, pharmacological stimulation or inactivation of metabolism may improve the metabolic fitness, survival, and anticancer activity of immune cells (137).

## Conclusions

**Figure 8:**
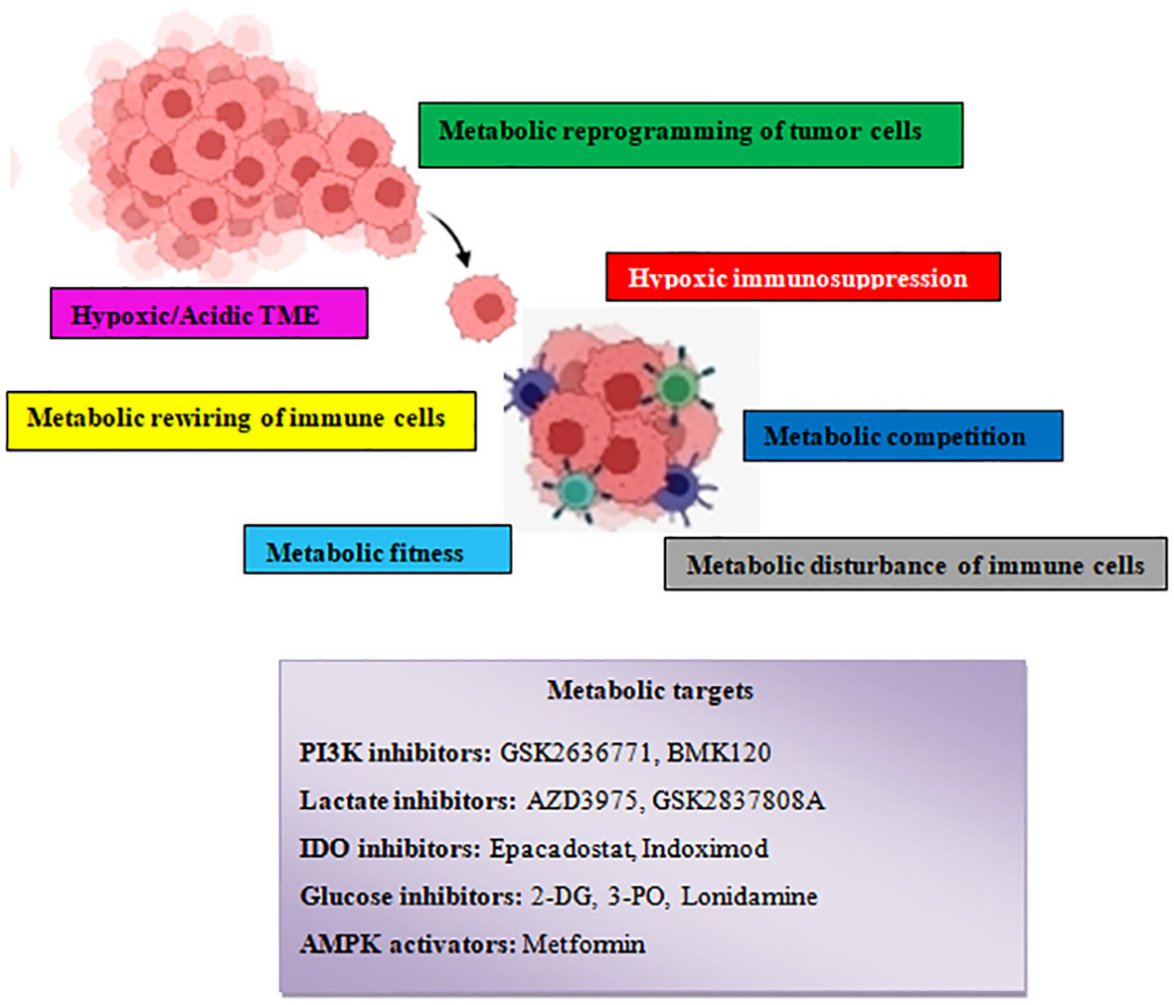
Schematic illustration showing the various tumor–driven immune metabolic reprogramming mechanisms during metastatic carcinogenesis and several therapeutic agents that target metabolic reprogramming signaling routes.

It is clear that tumor or immune cell metabolism targeting can work in conjunction with antitumor immunity. Immunotherapies frequently yield modest response rates; however, understanding and using metabolic interaction between tumor cells and immune cells has the potential to improve these rates (**Figure 8**). Although different combinations of metabolic agents and immunotherapies are already being tested in clinical trials, more research is needed to fully understand the metabolic mechanisms of immune evasion by tumors and the metabolic requirements of immune cells. Notably, metabolic programming of tumor cells not only influences immune cell antigen presentation and detection, but it may also change immune cell activity, thereby changing the immune response to tumors. Therefore, metabolic treatments may widen the range of malignancies that may be successfully treated with immunotherapy by increasing both the immunogenicity of cancer cells and immune cell responses against highly immunogenetic tumors.

## Author contributions

TK: Conceptualization, Writing – review & editing. SP: Supervision, Writing – original draft. DA: Data curation, Writing – review & editing. CC: Methodology, Writing – review & editing. IC: Methodology, Writing – review & editing. GF: Formal Analysis, Writing – review & editing. PE: Writing – review & editing. PZ: Project administration, Writing – review & editing. CKou: Project administration, Writing – original draft. CS: Validation, Writing – original draft. KP: Validation, Writing – review & editing. MD: Formal Analysis, Writing – review & editing. VK: Resources, Writing – review & editing. NV: Visualization, Writing – review & editing. VT: Visualization, Writing – review & editing. CM: Resources, Writing – review & editing. KV: Software, Writing – review & editing. SK: Visualization, Writing – review & editing. NK: Data curation, Writing – review & editing. KF: Resources, Writing – review & editing. CKos: Funding acquisition, Supervision, Writing – original draft.
